# Broad Ligament Fibroid Mimicking an Ovarian Tumor: A Case Report

**DOI:** 10.7759/cureus.113977

**Published:** 2026-08-04

**Authors:** Ravi S Ghuge, Kajal Mitra, Prashant Onkar, Suresh Phatak, Pranit B Pantawane

**Affiliations:** 1 Radiodiagnosis, NKP Salve Institute of Medical Sciences and Lata Mangeshkar Hospital, Nagpur, IND

**Keywords:** adnexal mass, broad ligament leiomyoma, cystic degeneration, extrauterine fibroid, magnetic resonance imaging

## Abstract

Broad ligament leiomyomas are rare extrauterine smooth muscle tumors that can mimic adnexal neoplasms because of their atypical location and degenerative changes. We report the case of a 47-year-old multiparous woman with lower abdominal pain for five to six months, worsening over the preceding eight to 10 days. Ultrasonography demonstrated a large predominantly solid-cystic abdominopelvic mass with minimal vascularity, with neither ovary separately visualized. Magnetic resonance imaging (MRI) revealed a well-defined mass centered in the right broad ligament region, showing a T2-hypointense solid component, cystic degeneration, mild progressive enhancement, and no diffusion restriction; the right ovary was not separately identified, raising suspicion of an ovarian neoplasm. Exploratory laparotomy demonstrated a well-encapsulated right broad ligament mass separate from the uterus, with compression but preservation of the right ovary. The mass was excised with preservation of the uterus, both ovaries, and ureter. Histopathology confirmed a benign leiomyoma with low mitotic activity and absence of coagulative tumor cell necrosis. This case highlights the importance of recognizing characteristic imaging features and anatomical relationships in differentiating broad ligament leiomyomas from ovarian neoplasms.

## Introduction

Leiomyomas are the most prevalent benign tumors of the uterus, originating from smooth muscle cells of the myometrium, and predominantly affect women during their reproductive years. Based on their relationship to the uterine wall, they are categorized as submucosal, intramural, or subserosal in location [1,2]. While subserosal fibroids may extend laterally, true leiomyomas arising primarily from the smooth muscle fibers of the broad ligament are distinctly uncommon and constitute a rare subset of extrauterine fibroids [3]. Clinical manifestations vary according to lesion size and anatomical location and may include menstrual disturbances, pelvic discomfort, or pressure-related symptoms due to involvement of adjacent pelvic organs [4]. As leiomyomas enlarge, they often undergo secondary degenerative changes such as hyaline, cystic, or myxoid degeneration, which can significantly modify their imaging appearance and complicate radiological diagnosis [5]. Broad ligament fibroids may represent either true tumors arising within the ligament or uterine fibroids that secondarily grow into the broad ligament space [3,6]. Their unusual location can result in distortion of normal pelvic anatomy, commonly displacing the uterus medially and the ureters laterally or inferiorly, occasionally leading to urinary tract or bowel symptoms [7]. Imaging typically reveals a well-defined pelvic mass with characteristics similar to uterine leiomyomas, and identification of vascular connections to the uterus along with separate visualization of both ovaries is crucial for differentiation from adnexal tumors [3].

## Case presentation

A 47-year-old multiparous woman presented with a five- to six-month history of gradually progressive dull lower abdominal pain, with worsening of pain over the preceding eight to 10 days. There was no associated history of urinary or bowel complaints, weight loss, anorexia, or fever. She reported secondary amenorrhea for the preceding eight months. General examination and vital parameters were within normal limits. Abdominal examination revealed a firm abdominopelvic mass corresponding to a 24-week gravid uterus. The mass was non-tender, showed restricted mobility, and its inferior margin could not be clearly defined.

On speculum examination, the cervix appeared healthy but was difficult to visualize owing to mass effect. Bimanual examination demonstrated obliteration of the vaginal fornices, with the uterus and adnexa not separately appreciable from the mass. Routine laboratory investigations were within normal limits. Serum cancer antigen 125 (CA-125) was 7.58 U/mL (reference range: 0-35 U/mL), cancer antigen 19-9 (CA 19-9) was 7.14 U/mL (reference value: <37 U/mL), and lactate dehydrogenase (LDH) was 210 U/L (reference interval: 84-246 U/L), all of which were within their respective reference ranges.

**Figure 1:**
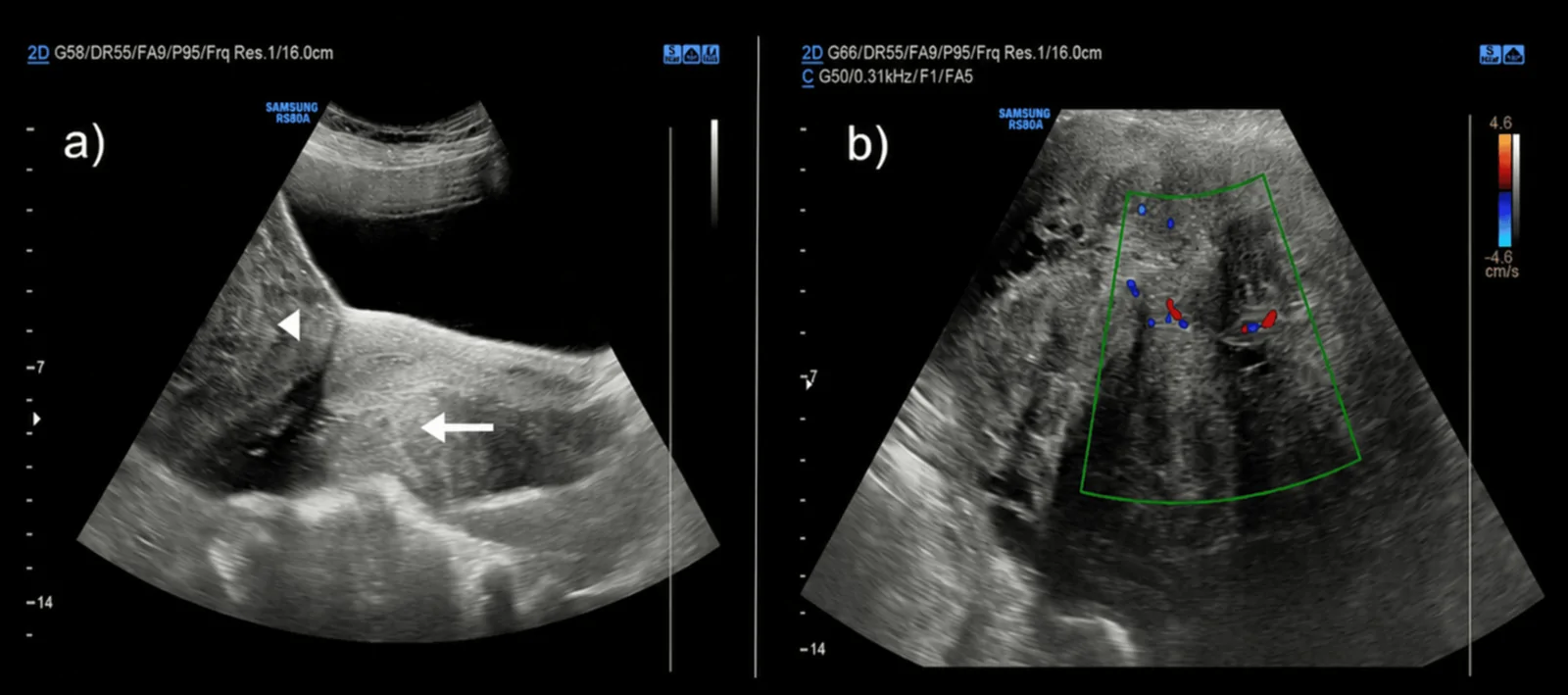
Ultrasonography findings (a) Gray-scale ultrasound image showing a large, heterogeneous, predominantly solid abdominopelvic mass with internal cystic areas adjacent to the uterus (arrowhead), with the uterus seen separately from this lesion, indicated by the arrow. (b) Color Doppler image demonstrating mild internal vascularity within the predominantly solid mass.

Transabdominal ultrasonography demonstrated a large heterogeneous, predominantly solid abdominopelvic lesion measuring 19.5 × 11.4 × 18.1 cm, with a few internal cystic areas, the largest measuring 7.9 × 2.7 cm (Figure 1a). No calcification was identified, and minimal internal vascularity was noted on color Doppler imaging (Figure 1b). The lesion was seen adjacent to the uterus, which appeared separate from the mass (Figure 1a). Neither ovary could be separately visualized on ultrasonography, raising the possibility of an ovarian neoplasm (Figures 1a, 1b).

**Figure 2:**
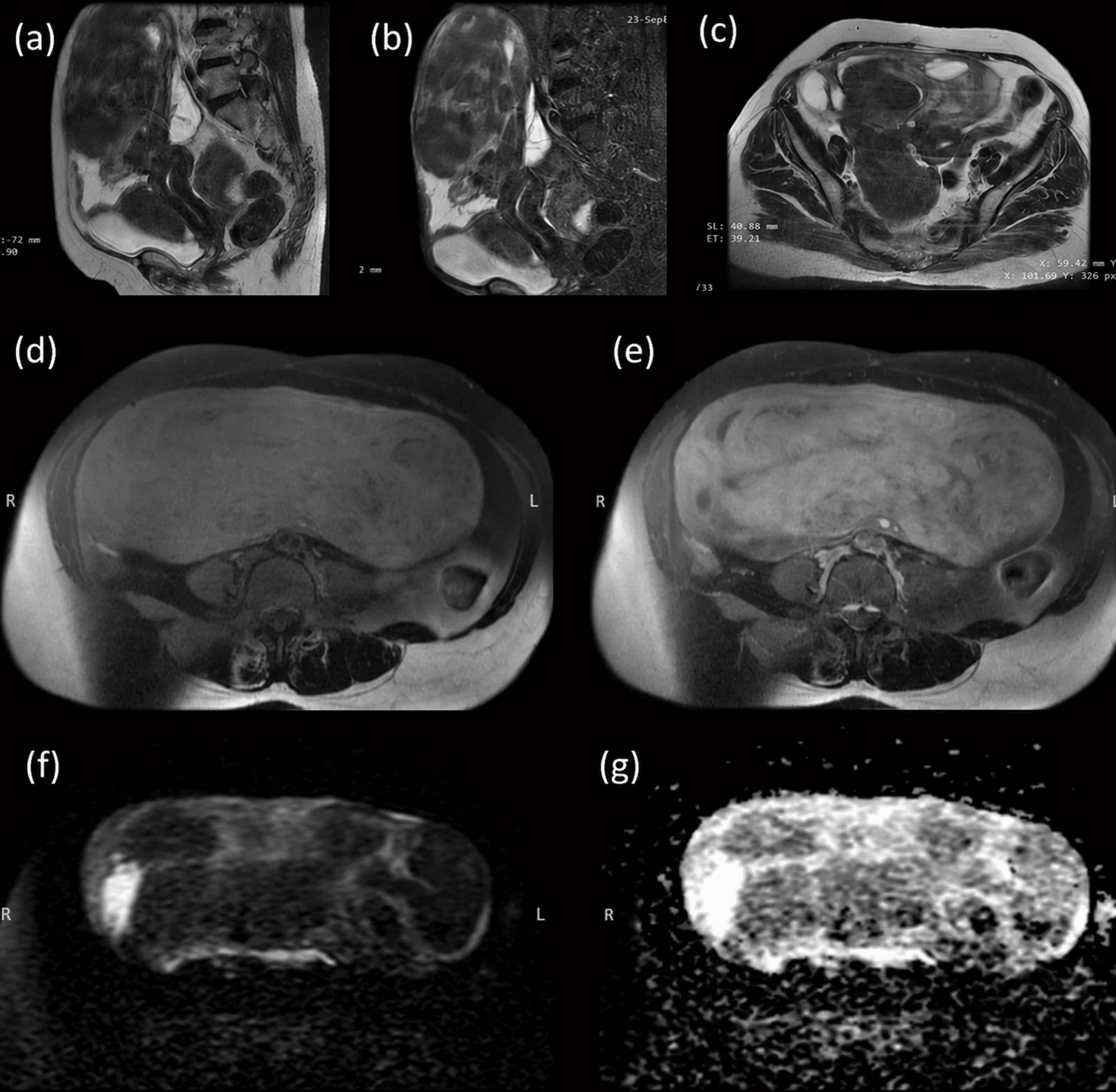
Magnetic resonance imaging findings (a,b) Sagittal T2-weighted images demonstrate a large, well-circumscribed, multilobulated, predominantly solid pelvic mass with areas of cystic degeneration. (c) Axial T2-weighted image showing the mass occupying the pelvis and displacing adjacent pelvic structures. (d) Axial T1-weighted image showing predominantly isointense signal within the solid component. (e) Post-contrast T1-weighted image demonstrating mild gradual enhancement of the solid component. (f) Diffusion-weighted image and (g) corresponding apparent diffusion coefficient map showing no diffusion restriction.

Magnetic resonance imaging (MRI) revealed a large, well-defined, multilobulated solid-cystic abdominopelvic mass measuring 8 × 21.4 × 17.4 cm in the anteroposterior, transverse, and craniocaudal dimensions, respectively, with an approximate volume of 1489 cc. The lesion was centered in the right pelvis, in the region of the broad ligament, and extended from the level of the pubic bone superiorly to the inferior border of the L2 vertebra. Sagittal T2-weighted images demonstrated a predominantly solid mass with multiple internal areas of cystic degeneration extending superiorly into the abdomen (Figures 2a, 2b). Axial T2-weighted imaging demonstrated the large pelvic component of the lesion and its relationship to the adjacent pelvic structures (Figure 2c). The solid component appeared isointense on T1-weighted images (Figure 2d) and markedly hypointense on T2-weighted and short tau inversion recovery (STIR) sequences (Figures 2a-2c), whereas the cystic areas were hypointense on T1-weighted images and hyperintense on T2-weighted/STIR images (Figures 2a-2d). Post-contrast T1-weighted images demonstrated mild progressive enhancement of the solid component on the venous phase (Figure 2e). Diffusion-weighted imaging (DWI) and the corresponding apparent diffusion coefficient (ADC) map showed no diffusion restriction within the solid component (Figures 2f, 2g).

The lesion maintained preserved fat planes with the adjacent structures. Sagittal images demonstrated its superior abdominal extension and mass effect on the uterus and urinary bladder (Figures 2a, 2b), while axial images demonstrated its relationship to the pelvic structures (Figure 2c). Anteriorly, the lesion abutted the anterior abdominal wall; laterally, it displaced the bowel loops laterally and posteriorly; posteriorly, it caused retroversion of the uterus, indented the rectum and sigmoid colon, and abutted the lower lumbar vertebrae and distal abdominal aorta. Inferiorly, it compressed the superior wall of the urinary bladder. The ureter was displaced posterolaterally by the lesion without evidence of upstream hydroureteronephrosis. The uterus was retroverted and appeared separate from the mass. The left ovary was separately visualized on MRI, whereas the right ovary could not be separately identified from the lesion (Figure 2c). No definite uterine attachment, vascular pedicle, claw sign, or bridging vessel sign connecting the mass to the uterus was identified on imaging. Although the lesion was centered in the region of the right broad ligament, non-visualization of the right ovary and absence of a definite uterine attachment raised the possibility of a right ovarian origin on preoperative imaging.

Exploratory laparotomy was performed. Intraoperatively, a large, well-encapsulated, lobulated mass was identified arising from the right broad ligament and was clearly separate from the uterus. These operative findings supported its classification as a true broad ligament leiomyoma rather than a uterine leiomyoma extending secondarily into the broad ligament. The uterus was preserved. The left ovary was normal and separately identified, whereas the right ovary was compressed by the mass but was preserved. The ureter was identified and preserved during surgery. The mass was excised with preservation of the uterus and both ovaries. No intraoperative complications occurred.

**Figure 3:**
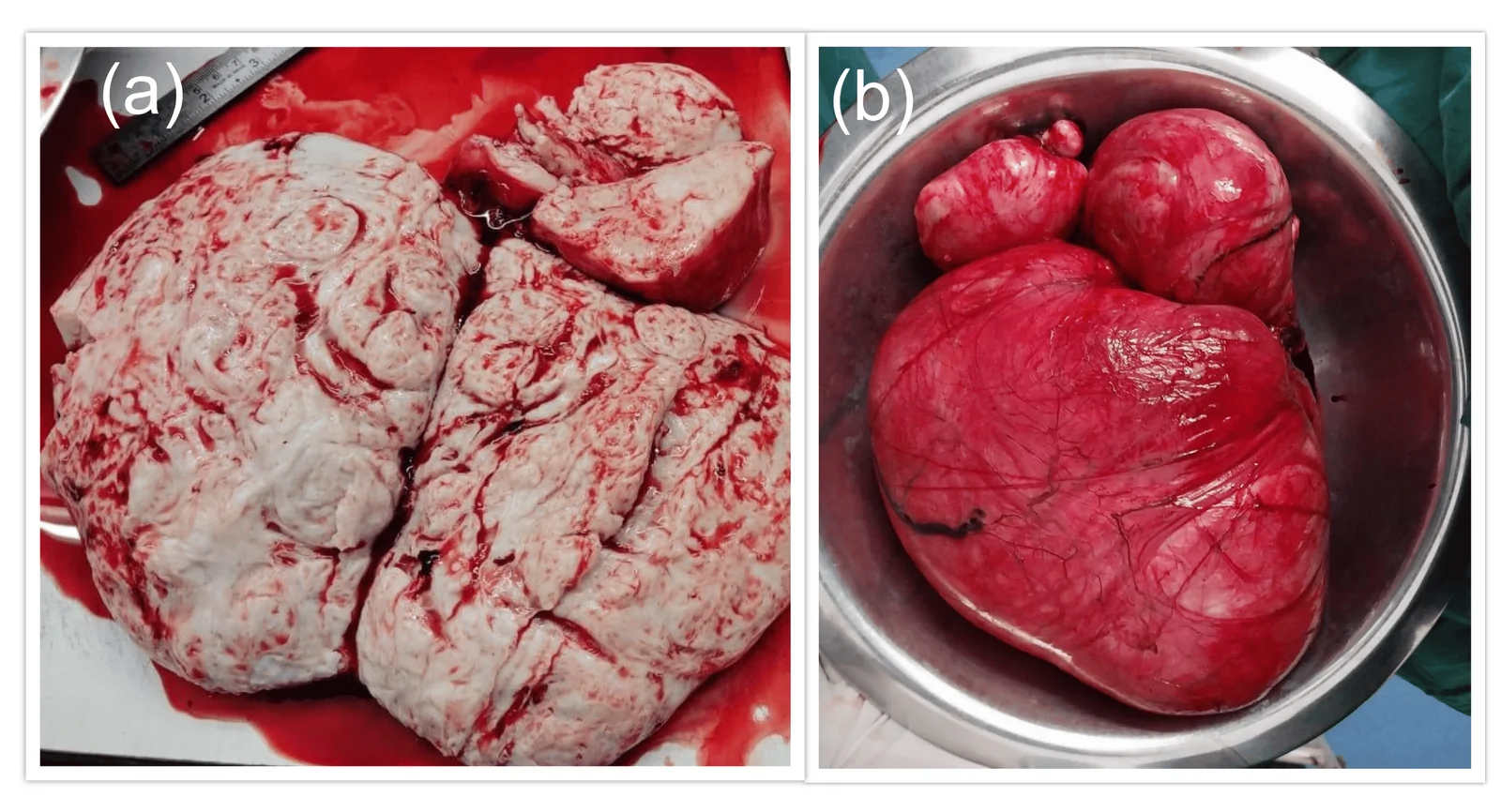
Intraoperative findings (a) Cut section of the excised broad ligament mass showing a characteristic pale white, firm, whorled appearance with focal areas of congestion and cystic degeneration, consistent with leiomyoma. (b) Gross specimen photograph demonstrating a large, well-circumscribed, multilobulated broad ligament leiomyoma with a smooth external surface and prominent congested serosal vessels following surgical excision.

The excised specimen was multinodular with a smooth external surface and prominent congested serosal vessels and gross examination of the cut surface demonstrated a firm, pale white, whorled appearance with focal areas of congestion and degeneration (Figures 3a, 3b). The gross degenerative areas corresponded to the cystic components demonstrated on ultrasonography and MRI, providing radiological-pathological correlation.

The postoperative course was uneventful, with no postoperative complications, and the patient was discharged on postoperative day 10. 

**Figure 4:**
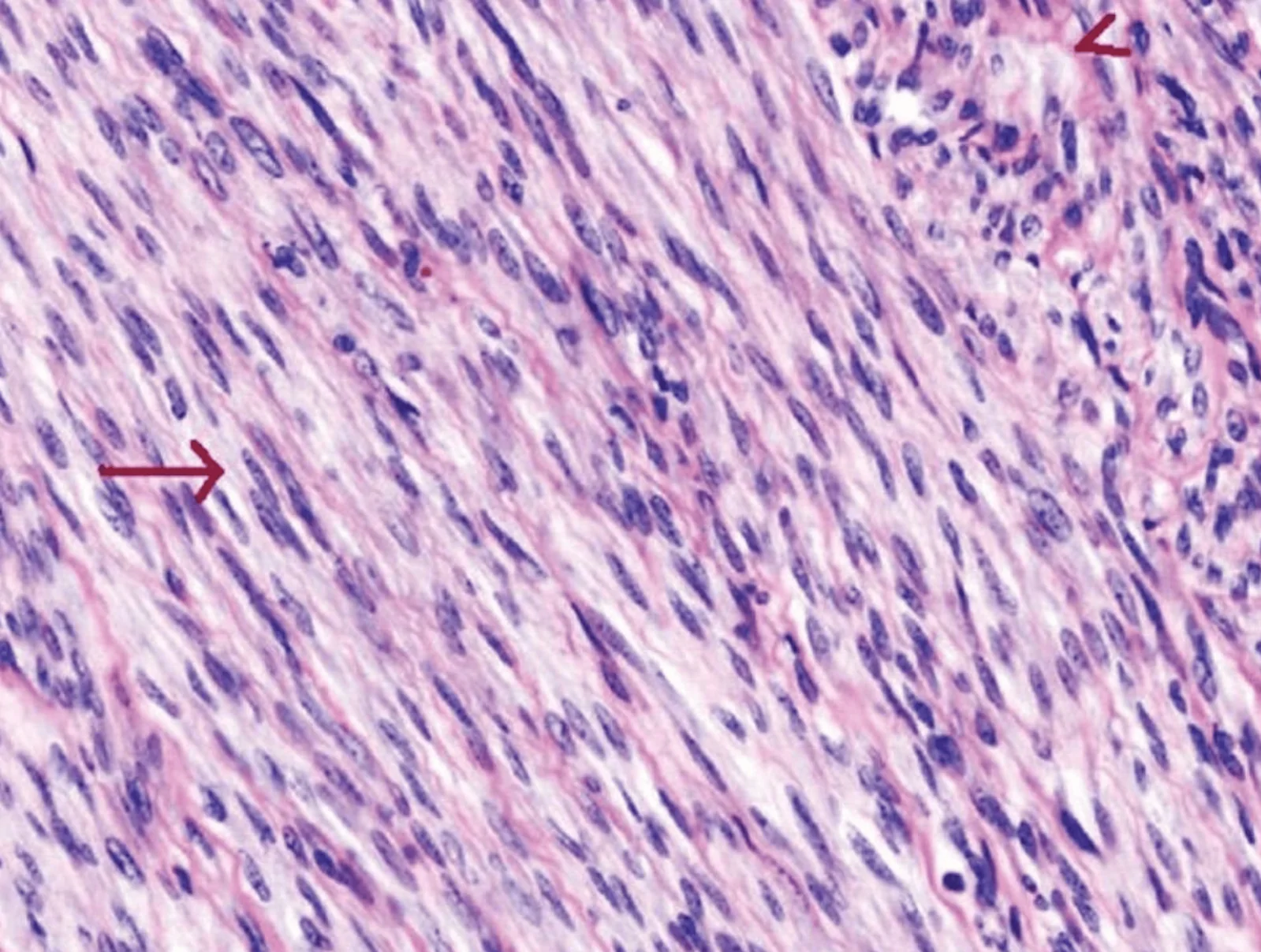
Histopathological findings The photomicrograph demonstrates intersecting fascicles of benign spindle-shaped smooth muscle cells (arrow) arranged in a characteristic whorled pattern. The cells possess elongated, blunt-ended (“cigar-shaped”) nuclei with finely dispersed chromatin and eosinophilic cytoplasm. No significant nuclear atypia, coagulative tumor cell necrosis, or increased mitotic activity is identified. Focal areas of hyalinization and sparse inflammatory cell infiltrate are present (arrowhead). These findings are consistent with a benign leiomyoma.

Histopathological examination demonstrated interlacing fascicles of benign spindle-shaped smooth muscle cells without significant nuclear atypia. Mitotic activity was low (five mitoses per 10 high-power fields), and coagulative tumor cell necrosis was absent, supporting the diagnosis of a benign broad ligament leiomyoma (Figure 4). Immunohistochemistry was not performed because routine histomorphological findings were considered sufficient to establish the diagnosis.

## Discussion

Broad ligament leiomyomas may occur as true extrauterine tumors or as parasitic subserosal fibroids that acquire an auxiliary blood supply from surrounding structures [6]. Their clinical presentation is largely influenced by mass effect, with ureteric compression being a recognized complication that can result in hydroureteronephrosis [7]. In the present case, operative demonstration of a right broad ligament origin separate from the uterus established the lesion as a true broad ligament leiomyoma, while ureteric displacement without obstruction reflected its substantial mass effect.

On ultrasonography, leiomyomas generally appear as well-defined solid masses with a characteristic whorled pattern; however, degenerative changes may alter echotexture and mimic adnexal pathology [2,5]. MRI is particularly valuable in distinguishing broad ligament fibroids from adnexal masses by accurately delineating pelvic anatomy and providing superior soft tissue characterization [3]. Typical leiomyomas demonstrate low signal intensity on T2-weighted images, while areas of cystic or myxoid degeneration appear hyperintense [5]. In this case, the low T2 signal of the solid component, cystic degeneration, mild progressive enhancement, and absence of diffusion restriction favored a benign smooth muscle tumor; however, non-visualization of the right ovary and absence of a definite uterine attachment raised the possibility of an adnexal origin.

The differential diagnosis includes ovarian fibroma or fibrothecoma, pedunculated subserosal uterine fibroid, leiomyosarcoma, broad ligament schwannoma, lymphadenopathy, and solid ovarian malignancy [3,5]. Ovarian fibroma or fibrothecoma typically presents as a solid adnexal mass with markedly low T2-weighted signal intensity and minimal enhancement, often without separate visualization of the ovary. A pedunculated subserosal uterine fibroid should be considered when continuity with the uterus and bridging vessels are identified on Doppler or MRI. Leiomyosarcoma is suggested by a large, heterogeneously enhancing mass with irregular margins, internal hemorrhage or necrosis, and rapid growth. Broad ligament schwannoma usually appears as a well-defined mass without uterine attachment and may demonstrate a target sign with cystic degeneration on MRI. Lymphadenopathy commonly manifests as multiple rounded, homogeneous soft tissue masses lacking a whorled architecture or calcification. Solid ovarian malignancy should be suspected when a solid, enhancing adnexal mass is associated with ascites, peritoneal deposits, and elevated tumor markers such as CA-125.

Applied to the present case, ovarian fibroma/fibrothecoma remained a consideration because of the low T2 signal and non-visualization of the right ovary, whereas the degenerative pattern favored leiomyoma. The absence of uterine attachment, claw sign, or bridging vessels argued against a pedunculated subserosal fibroid. The well-defined margins, preserved tissue planes, mild progressive enhancement, absence of diffusion restriction, and normal tumor markers reduced suspicion for leiomyosarcoma or an aggressive ovarian neoplasm. Schwannoma and lymphadenopathy were less favored because typical neural tumor morphology and nodal distribution, respectively, were absent.

Importantly, intraoperative identification of the right ovary compressed by, but separate from, the mass established that its non-visualization on imaging was due to anatomical distortion rather than ovarian origin. Radiological-pathological correlation was concordant, with the cystic imaging components corresponding to gross degenerative areas; routine histomorphology confirmed benign leiomyoma and was considered sufficient without immunohistochemistry.

## Conclusions

Broad ligament leiomyomas are rare entities that may pose significant diagnostic challenges due to their proximity to adnexal structures and variable imaging appearances. Familiarity with their characteristic imaging features, particularly on MRI, is essential to avoid misdiagnosis. Broad ligament leiomyoma should be included in the differential diagnosis of adnexal masses, particularly when the organ of origin cannot be confidently established on imaging. In the present case, the lesion demonstrated a large, predominantly solid pelvic mass with cystic degeneration and an inability to separately identify the right ovary on imaging, resulting in an initial radiological impression of an ovarian neoplasm. However, the characteristic low T2 signal intensity of the solid component, mild progressive enhancement pattern, and absence of diffusion restriction were more suggestive of a leiomyomatous origin. Surgical and histopathological findings subsequently confirmed the diagnosis of a broad ligament leiomyoma.
